# Dr. Clark's Vindication of British Medicine

**Published:** 1824-06-01

**Authors:** 


*Lettera del Dottore* GIACOMO CLARK *al Professore* GIACOMO TOMMASINI *intorno alla Letteratura Medica Inglese.* Pp. 47. Roma 1823.

IN our Number for June 1822, we noticed, with commendation, Dr. CLARK's *Italian* defence of the Edinburgh School of Medicine, against the attack made on it, or rather against the erroneous view given of it, by the celebrated Bolognese professor, Tommasini. In this vindication of his alma mater, Dr. CLARK incidentally noticed Tommasini's very imperfect acquaintance with the medical literature of this country, as evinced in several parts of his discourse, observing that, if he (Tommasini) had been better acquainted with our *good* authors, he would have formed a higher opinion of British Medicine, both scientific and practical. This insinuation of defective knowledge of a matter, respecting which it would have been, indeed, wonderful if his knowledge had not been defective,—seems to have considerably excited the personal and patriotic feelings of the Professor, who forthwith indites and publishes a Reply to Dr. Clark, intended, at once, to be a justification of his former statements, and a further and more decisive proof of his acquaintance with our medical literature. As might have been expected, he failed egregiously in the latter particular; and quite as much, or more so, in respect of the authors cited, as of those omitted by him. As, however, this pretended account of our medical literature, and the view of our medical doctrine and practice thence derived, presented the semblance, at least, of being founded on fact,—was the deliberate and avowed production of a man justly esteemed for his talents and extensive erudition, and who, moreover, might naturally be considered as peculiarly qualified for the task he had undertaken, on account of his recent visit to England,—and, finally, as it was submitted to readers who were at least as ignorant of the subject as the author;—Dr. Clark thought it necessary once more to break a lance with the redoubted Italian, and to attempt to do that for British medicine in general, which he had already so successfully done for the clinical school of Edinburgh. This attempt is now before us in the form of an *Italian* letter, of 47 pages, addressed to Tommasini; and we must say, after an attentive perusal, that the essay has been most successful, the author having acquitted himself in a manner infinitely creditable to him as a gentleman, a scholar, and a British physician. In this little work we scarcely know which most to admire, the author's thorough knowledge of his subject and complete discomfiture of his opponent, or the gentle yet firm and dignified tone in which his object is accomplished. Indeed, we do not remember to have read any thing of a controversial nature that left us less to regret, both in matter and manner, than the pamphlet before us; and while we congratulate the author on such honourable testimonies of his temper, his talents, and his learning, we congratulate ourselves and our professional brethren in this country, on having so able an advocate, of their cause, in the field of foreign literature.

As the little work of Dr. Clark is, nevertheless, interesting to us rather as a literary curiosity, than as containing matters calculated to instruct the well-informed members of the profession in this country, we shall content ourselves, in this place, with a few brief notices of its more important contents, taken at random from its pages.

In our review of Dr. Clark's former letter, we gave a detailed account of that most valuable institution the Clinical School of Edinburgh. The same subject is again slightly touched on in the present letter. Dr. Clark readily concedes to Tommasini, that, in some respects, the Italian system of instruction is more favourable for the student, but that it is certainly less humane towards the patient. The system of *lecturing* (for such it is) on the disease, in the presence of the patient is, unquestionably, cruel and disgusting in the highest degree; and, as Dr. C. observes, would not be submitted to by the inmates of a British hospital.

"I have myself (says Dr. C.) heard this done at the bedside of a poor *consumptive* patient; and a friend of mine was lately present when the same practice was adopted in a case of *cancer uteri:* at the end of the exposition, the poor woman burst into a flood of tears." And, truly, no wonder! Dr. Clark, however, admits that another branch of the Italian method—that, namely, of consigning the treatment of patients, under the superintendence of the professor, to the more advanced students, might be adopted with much advantage in this country. In this we entirely agree with him.

It has long been a common subject of complaint with the medical writers of this country, that their discoveries and improvements were overlooked or disregarded by continental authors, more especially the French, until such time as they had acquired a sort of right and title, by adoption, transformation, or intermarriage, to be considered as legitimate productions of their *foster-land.* Then, indeed, they never failed to be introduced to the savans of their new country, with all the pride, pomp, and circumstance to which they were intrinsically and extrinsically entitled. It is true that similar complaints were made, and still continue to be made, of us, on the other side of the channel; we think, however, (in all candour and honesty,) with decidedly less reason on the part of our accusers. And we think the difference in this particular may be very simply and satisfactorily explained, without any illiberal or offensive reference to national character, by the mere fact of the languages of the countries alluded to being much better, and much more generally understood in England, than our language is on the Continent. We think it must be admitted by every one capable, from personal observation, of judging, that for one Frenchman who understands English, there are twenty Englishmen who understand French. The same is true, but in a lesser degree, of the Italians and English; and, in a still lesser degree, of the Germans and English. Be this as it may, it is sufficiently certain that the French and Italian medical authors betray lamentable ignorance of our literature. A very striking and indeed amusing instance of this is now before us in the letter of Tommasini (one of the best informed foreigners, be it remembered) to Dr. Clark. In his first letter, Dr. C. as we have already remarked, expressed his opinion of his opponent's imperfect acquaintance with our best authors (naming several of these.) Tommasini, in his reply, not only admits the full merit of the writers cited by Dr. C. but adds several others, equally meritorious, not cited by him; but with the following countervailing stigma: "To these authors (says he) I could oppose too great a number of authors equally modern, but of quite a different stamp;" and then subjoins the following selection of authorities, e. g. "*Raven* (on Cholchicum in Hysteria, &c.) *Gaikskell* (on Nux Vomica in Palsy, &c.) *Jutliffe* (on Opium in Tetanus,) *Hutchinson* (on Tic Douloureux,) *Dewees* (on Guaiacum in Amenorrhœa,) *Sameson* and *Gough Parker* (on the nitrate of Silver in Epilepsy,) *Hamen* (on Blood-letting in Hydrocephalus,) *Witlam* (on Cicuta,) *Wansbrough* (on Digitalis in Phthisis. &c.) *Copland* (on Chorea,) and *Willan* (on Cutaneous Diseases.)"

Without seeing it, one could scarcely believe that such an instance of ignorance could attach to the character of a meritorious and learned author. The lesson it ought to teach, however, is, not to despise our neighbours, but to make us more cautious than we are, in deciding on the true merits of foreign writers, and on the general spirit of foreign literature and science. Well might Dr. Clark say, on perusing the above list, that "the mere inspection of the *names* of these *authors* suffices of itself, without further argument, to show the slight value of opinions founded on such authorities;" and we are not surprised to find that, with the exception of those of Drs. Willan and Copland, the *names* even of these *authors* were unknown to Dr. Clark, and that he was forced to trouble his friends in England, more than once, before he could gain any intelligence of them! The truth is, that Tommasini's materials were principally derived from our periodical journals; and that he was either unable or unwilling to discriminate between the crude effusions of the ignorant student, and the matured labours of learning and experience.

This accounts not only for the particular selection made, but for the mixing up, in the same record, of names of just and respectable authority, with others of no authority at all. Indeed, several of the names quoted have, we are quite sure, no substantial bearers in this country; and we sincerely regret that nor head nor ears exist to be "ravished with the whistling of a name," or "damned to everlasting fame," in the erudite pages of the professor of Bologna.* Ignorance and error of a precisely similar kind are charged, by Dr. Clark, on Broussais, in his pretended examination of the medical doctrines of England, in which we find the names of obscure contributors to periodical journals making as conspicuous a figure as in the more temperate exposition of Tommasini. In joining with Dr. Clark in this unqualified condemnation of Broussais's View of British Medicine, which is truly *astonishingly* meagre, and, we may even add, contemptible—we think it necessary to join with him, also, in yielding our highest respect and regard for that distinguished author, and to admit, that we have derived much pleasure and profit from the examination, and, we will add, adoption (in part at least) of his original and profound pathological doctrines.

After exposing his opponent's ignorance of our medical literature by an examination of the authorities adduced by him, Dr. Clark proceeds to lay before him a correct view of the really standard works that have appeared in England during the present century. This list is neatly and clearly arranged under the heads of the diseases that affect the different organs; and includes the full titles of more than a hundred works, with concise remarks on the nature and character of several of them. We regret that our limits will not permit us to transfer to our pages this catalogue raisonnée of our recent medical authors; as we are convinced it would not be without its use to the junior members of the profession, even in this country;—as a standard of reference, and a guide for them to our recent literature, it must be of vast benefit to the physicians of Italy and France.

In combating the professor's charge of *empiricism* brought against our practical medicine, our author has many just and forcible observations which our limits forbid us to notice. We must content ourselves with a few detached extracts.

"It is indeed true," says Dr. Clark, "that in England we have no

* Like appreciations of British medical authority must be familiar to those much accustomed to the perusal of foreign authors. "Whence comes it," says a recent Italian writer, according to Dr. Clark, "whence comes it, that Buchan *and* Cullen are of a very different opinion?" *Buchan* and *Cullen !!* In another place Dr. C. remarks, "We observe in Italian medical books, from Borsieri downwards, *Buchan* cited as an authority; *Thomas* is at present quoted as a *celebrated author;* and, latterly, the book even of Reece himself *("di Reece stesso")* has been translated, doubtless under a false impression of its character." p 5. After *this last* piece of information, it would certainly be only wasting words to endeavour to confirm the belief of the false estimate of British medicine entertained by the Italians.

*general doctrine*—no grand and all-explaining theory; not, indeed, because there exists any dislike to rational theory, but because there exists a firm conviction that the degree of our knowledge is at present too limited to authorise the formation of a theory capable of explaining the intimate nature of our various diseases, and, still less, of explaining the mode of action of the medicines employed in their cure." 37.

"Although the term *diathesis* is not received in England in the same sense as in Italy, British practitioners are not, on this account, less desirous of distinguishing diseases according to their essential characters, or less diligent in their researches for their *true pathology;* nor are they backward in generalising their observations founded on such researches, and in applying the results to practice—provided always, that these results are based on a faithful induction of facts. When, however, this basis is wanting, as I apprehend it must frequently be to those who vaunt most of their theory—in this case, the English physician is not ashamed to return to empiricisms, which, however despised by modern theorists, we are obliged, nevertheless, humbly to confess to be, in the actual state of our knowledge, at once the best resource of the practitioner, and the best safeguard of the patient against the pernicious consequences of theory. And here I beg to be understood as speaking not of that blind empiricism which is the offspring of ignorance and presumption, but of that philosophical empiricism, which is the result of observation and sound experience. At the same time, I am far from embracing the cause of empiricism. I admit its utility only when we are abandoned by the light of a true pathology. I am even ready to confess that it has exercised, and still exercises an undue influence over the minds of many English practitioners. This, perhaps, may be justly considered as a natural consequence of the unsatisfactory results of all medical theories. That the better class, however, of British practitioners are influenced by a very different spirit, is sufficiently manifest from the works already quoted, &c. &c.

"That many works of a different character are to be found in England, I am far from denying; but, in every country, the number of insignificant is always greater than that of good writers." 39.

In reply to Tommasini's boast of the refutation of the Brunonian doctrine being left for foreigners, Dr. Clark gives the following home stroke to his antagonist.

"The so much vaunted glory of confuting the doctrines of the *Scottish Reformer*, is now right willingly abandoned by his countrymen, to the writers of those nations who were the victims of his errors. In Great Britain, at the time of the promulgation of the system of Brown, medical science was too much enlightened to be either seduced by its specious allurements, or led astray from the true path of observation, or cheated out of the experience of ages." 40.

In proof of the general spirit of zeal and intelligence with which medicine is at present cultivated in England, Dr. Clark refers to the vast extent of our medical publications, and the number and value of our periodical journals. We shall conclude our notice of this interesting publication with the author's brief comparison of the English and Italian schools.

"The labours of the Italian physician are conducted with the view of supporting a doubtful theory; those of the English, in the design of amassing materials whence may eventually be composed a theory that will not pass away. If the English school is characterised by a spirit of extreme caution in the admission of facts, and by a philosophy perhaps too strict, in deducing general principles from these: the Italian school appears to me to be characterised by too great a facility in admitting facts, and too great a precipitancy in deducing consequences. Which of these defects is most hurtful to the student, I leave others to decide: to me it appears that the effect of the *former* is to excite in the youthful mind a love for observation, a commendable diligence and circumspection in the examination of facts, and a philosophic caution in drawing conclusions; and that of the *latter*, rather to withdraw the attention from observation, and to supply the place of this with an overweening confidence in theory." 46.

We here take our leave of our able and excellent author, and beg to thank him, in the name of our profession and country, for the noble and triumphant manner in which, "in strands afar remote," he has defended the cause of British Medicine.

